# Sagittal plane knee kinematics can be measured during activities of daily living following total knee arthroplasty with two IMU

**DOI:** 10.1371/journal.pone.0297899

**Published:** 2024-02-15

**Authors:** Bradley M. Cornish, Laura E. Diamond, David John Saxby, David G. Lloyd, Beichen Shi, Jenna Lyon, Kevin Abbruzzese, Price Gallie, Jayishni Maharaj

**Affiliations:** 1 Griffith Centre of Biomedical and Rehabilitation Engineering, Menzies Health Institute Queensland, Griffith University, Gold Coast, Queensland, Australia; 2 School of Health Sciences and Social Work, Griffith University, Gold Coast, Queensland, Australia; 3 Stryker Corporation, Kalamazoo, Michigan, Unites States of America; 4 Coast Orthopaedics and Sports Medicine, Gold Coast, Queensland, Australia; Universita Politecnica delle Marche Facolta di Ingegneria, ITALY

## Abstract

Knee function is rarely measured objectively during functional tasks following total knee arthroplasty. Inertial measurement units (IMU) can measure knee kinematics and range of motion (ROM) during dynamic activities and offer an easy-to-use system for knee function assessment post total knee arthroplasty. However, IMU must be validated against gold standard three-dimensional optical motion capture systems (OMC) across a range of tasks if they are to see widespread uptake. We computed knee rotations and ROM from commercial IMU sensor measurements during walking, squatting, sit-to-stand, stair ascent, and stair descent in 21 patients one-year post total knee arthroplasty using two methods: direct computation using segment orientations (r_IMU), and an IMU-driven iCloud-based interactive lower limb model (m_IMU). This cross-sectional study compared computed knee angles and ROM to a gold-standard OMC and inverse kinematics method using Pearson’s correlation coefficient (R) and root-mean-square-differences (RMSD). The r_IMU and m_IMU methods estimated sagittal plane knee angles with excellent correlation (>0.95) compared to OMC for walking, squatting, sit-to-stand, and stair-ascent, and very good correlation (>0.90) for stair descent. For squatting, sit-to-stand, and walking, the mean RMSD for r_IMU and m_IMU compared to OMC were <4 degrees, < 5 degrees, and <6 degrees, respectively but higher for stair ascent and descent (~12 degrees). Frontal and transverse plane knee kinematics estimated using r_IMU and m_IMU showed poor to moderate correlation compared to OMC. There were no differences in ROM measurements during squatting, sit-to-stand, and walking across the two methods. Thus, IMUs can measure sagittal plane knee angles and ROM with high accuracy for a variety of tasks and may be a useful in-clinic tool for objective assessment of knee function following total knee arthroplasty.

## 1. Introduction

Total knee arthroplasty (TKA) is an effective surgical treatment for knee osteoarthritis (OA) that reduces pain, lessens functional impairments, and improves quality of life [1, 2]. Nevertheless, patient outcomes, including post-operative complication rates, knee function, and patient satisfaction can vary due to differences in surgical techniques, prosthetic design, and implant alignment [3, 4]. Recently, robotically assisted Total Knee Arthroplasty (TKA) has improved alignment of prosthetic implants, optimised ligament balancing, increased knee range of motion (ROM), and reduced surgical times [1, 5, 6]. The use of robots has led to lower levels of post-operative pain and analgesic use, greater improvements in pain at 6- and 12-months post-operation, lower episode-of-care costs, and greater patient satisfaction compared to standard surgical approaches [6–8]. However, accurately assessing post-operative functional outcomes remains difficult.

Post-operative evaluations include clinical assessments of function and ROM, patient reported outcome measures (PROMS), and radiographic assessment of implant position [7]. Though recommended, the use of quantitative functional-based measures is less common due to timely and costly procedures, and consequently, are often inferred from PROMS [9]. However, there is poor concurrent validity between PROMS and functional measures in people with TKA [9]. Patient reports may relate more to pain than performance in functional tests, are confounded by the function and strength of the uninvolved limb, and fail to detect changes in the affected limb post TKA [10]. Additionally, PROMS commonly exhibit a ceiling effect in which they do not reliably discriminate between moderate and excellent outcomes, as clustering occurs at the upper limit of scoring [11].

Quantitative measures of knee kinematics during walking and other activities of daily living (ADL) may better describe knee function and disease status following knee arthroplasty. Prior to surgery, individuals with knee osteoarthritis (OA) typically display less knee excursion during the loading phase of gait and when climbing stairs, with greater knee adduction angle throughout gait when compared to age-matched controls [12, 13]. Following knee arthroplasty, knee mechanics partially restore [14, 15], although functional deficits often remain when compared to healthy controls [16]. Post TKA, individuals typically show reduced knee excursion (i.e., lower functional range of motion) and reduced external knee flexion moments [17, 18], that may be attributed to reduced knee extensor strength [19]. Evaluating knee biomechanics post-operatively during TKA recovery may better describe post-operative knee function, objectively inform rehabilitation decisions, and lend itself to improved surgical techniques. In the case of robotically assisted TKA, objective data such as knee kinematics may be analysed with respect to surgical information such as implant alignment, implant type, ligament balancing, and surgical duration, which can be logged automatically by the robot systems [6].

Current methods for measuring knee kinematics are time-consuming, costly, require specialised equipment such as three-dimensional optical motion capture (OMC), and operator expertise [20]. Optical motion capture is largely unportable and involves lengthy setup and participant preparation that make it impractical for clinical or home use. Wearable sensors, such as inertial measurement units (IMU), may provide an affordable, portable, and practical solution to movement analysis outside the laboratory [21–23] that may inform clinical practice [24]. Inertial measurement units combine multiple sensors; typically an accelerometer, a gyroscope, and a magnetometer, into one device that measures orientation, rotations, and accelerations [25]. For individuals post total hip arthroplasty, IMU have been shown to detect improvements in gait following surgery and provide objective data to complement PROMs [26]. Additionally, a recent review suggests that gait parameters and joint kinematics measured using wearable sensors offer greater insight to functional recovery than traditional functional outcome measures such as the timed-up-and go, and static ROM tests [27].

Inertial measurement units have measured joint angles during functional tasks with root-mean-square-difference (RMSD) less than 5 degrees compared to OMC [22, 28–30], which were not different to OMC for measurement of knee ROM during walking in individuals post total hip arthroplasty [31]. However, few studies have validated their use during multiple ADL [32] in a clinical population. For those following TKA, IMUs have shown to measure knee flexion ROM with an average error of roughly 4 degrees during clinical tests [33] but was not assessed during functional tasks and ADL that may indicate a patient’s knee function. Evaluating the accuracy of IMUs across multiple ADL would provide support for their use as a clinical measurement tool and may identify tasks during which IMU measurements are accurate and acceptable for objective assessments of knee function in individuals post TKA.

The aim of this study was to compare knee kinematics using IMU sensors and OMC in individuals 1-year post robotically assisted TKA during walking, squatting, sit-to-stand, stair ascent, and stair descent tasks. Knee kinematics from IMU were calculated using an interactive lower limb model (m_imu) and from raw quaternions (r_imu) measured using commercial IMU sensors (Notch Interfaces Inc. New York, USA). We hypothesised that knee angles for the m_IMU would have lower RMSD and higher correlation (R) than the r_IMU when compared to OMC. Furthermore, we hypothesised that RMSD in the sagittal plane would be lower than out-of-plane angles for all activities. We hypothesised that the sagittal plane knee ROM calculated using m_IMU would have smaller differences t compared to OMC than r_IMU.

## 2. Methods

### 2.1 Participants

Twenty-one individuals (9F/12M; mean ± standard deviation; age: 69 yrs ± 8; height: 1.71 m ± 0.11; body mass: 93.89 ± 24 kg) one year post robotically assisted TKA were recruited for this study between August and December 2020. All surgeries were performed by a single surgeon (PG), using the MAKO system (Stryker, Michigan, USA). Participants were included if they were twelve months post Mako assisted TKA, were older than 18 years, and self-reported their ability to walk for 30 minutes continuously. Participants were excluded if they were unable to walk independently; had any other serious lower limb injury in the past twelve months; had muscle degenerative disorders or motor impairments; had a body mass index greater than 40. Ethical approval was obtained from the Griffith University Research Ethics Committee and participants provided their written informed consent prior to testing.

### 2.2 Experimental protocol

Participants were asked to complete five tasks, in a fixed order: (i) walking on level-ground; (ii) bilateral squatting to max depth with arms crossed over chest; (iii) sit-to-stand from chair (44cm) with arms folded over chest and without use of arm rests; (iv) stair ascent; and (v) stair descent. Participants were given time to familiarise with each task, with all movements completed at a self-selected pace. Each participant was asked to perform 3 practice repetitions followed by a short break (1 to 2min) and 5 recorded repetitions of each task, with breaks (10 to 20min) between tasks to reduce fatigue. To allow for automatic calculation of joint kinematics, the Notch sensor system was calibrated according to manufacturer instructions by instructing the participants to stand stationary in a neutral reference posture, prior to each activity. For scaling of the OpenSim model, marker positions were collected by the OMC system from the participant in a static relaxed standing trial.

### 2.3 Experimental setup

**Fig 1:**
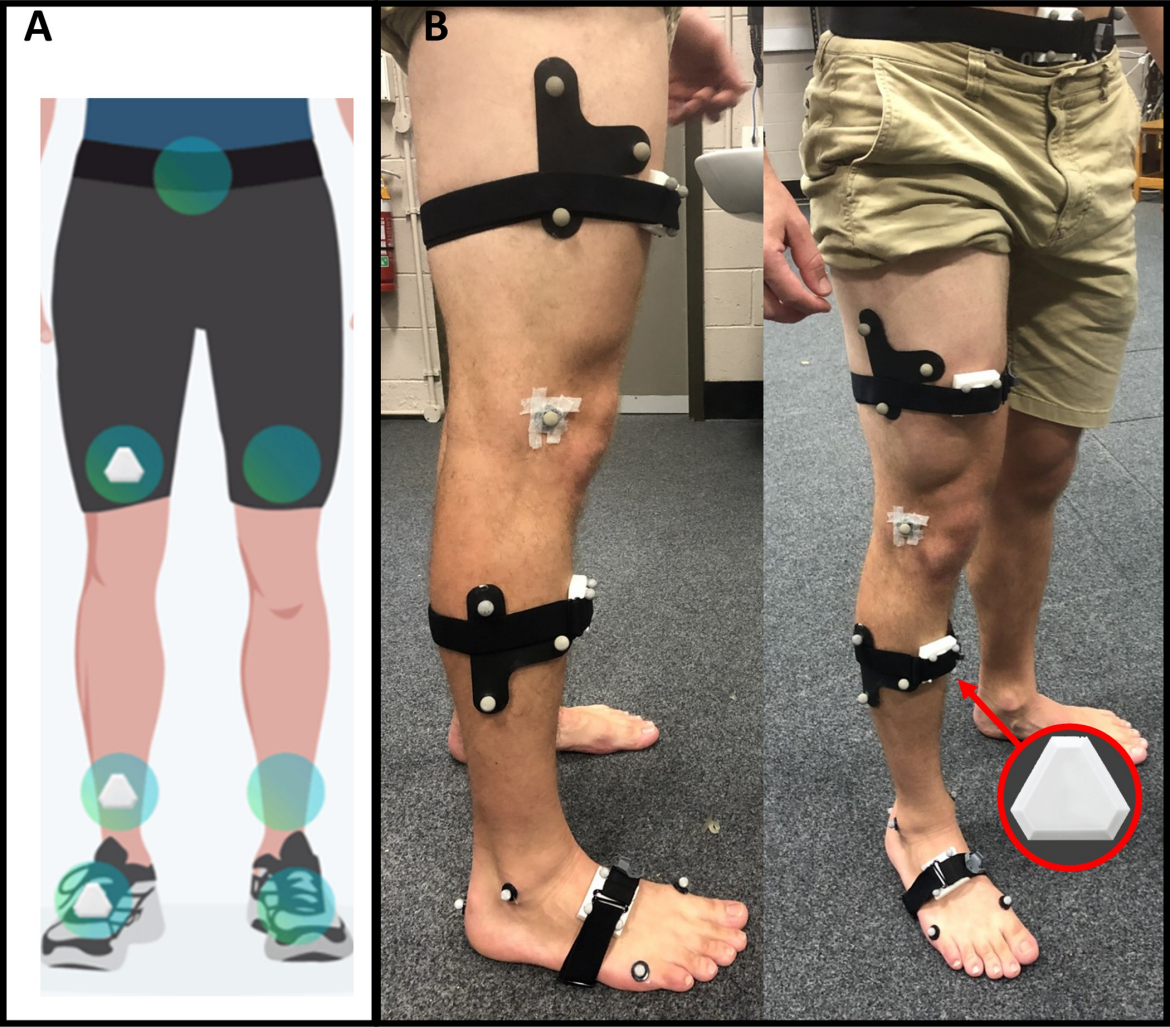
Participant preparation. A. Schematic showing the placement IMUs on body segments based according to manufacturer recommendations B. Images illustrate the methodological setup used to simultaneously measure knee kinematics from inertial measurement units (IMUs) placed on the thigh, shank, and foot and an optical motion capture (OMC) system.

A 10-camera Vicon OMC system (Vicon, Oxford, UK) was used to collect marker trajectories (200Hz) from 17 retro-reflective markers placed on the pelvis, thigh, shank, and foot of the affected limb (Fig 1). The marker set consisted of a three-marker cluster on the thigh and shank segments and anatomical markers placed over the right and left anterior-superior iliac spines; right and left posterior-superior iliac spines; medial and lateral femoral epicondyles; medial and lateral malleoli; posterior aspect of the foot and the first and fifth metatarsal heads. Ground reaction forces (1000Hz) were recorded for level overground-ground walking, squatting, and sit-to-stand activities using 3 force plates (510 mm × 465 mm, AMTI, USA) embedded into the ground and arranged in series in the centre of a walkway, approximately 10-metres long. For stair-ascent and stair-descent tasks, ground reaction forces (1000Hz) were recorded using a force-instrumented staircase with 3 force-plate embedded within the first three stairs (Bertec Corporation, Columbus, OH).

Segment rotations and orientations (200Hz) were recorded simultaneously to the OMC and ground reaction forces using IMUs, which contained an accelerometer, a gyroscope, and a magnetometer (Notch Interfaces Inc. New York, USA). The IMUs were affixed to the pelvis, thigh, shank, and foot of the affected limb using double sided tape and Velcro straps, following manufacturer instructions with devices placed on the anterior portion of each segment oriented upwards (Fig 1). IMU data were collected simultaneously with the OMC system.

### 2.4 Data processing

**Fig 2:**
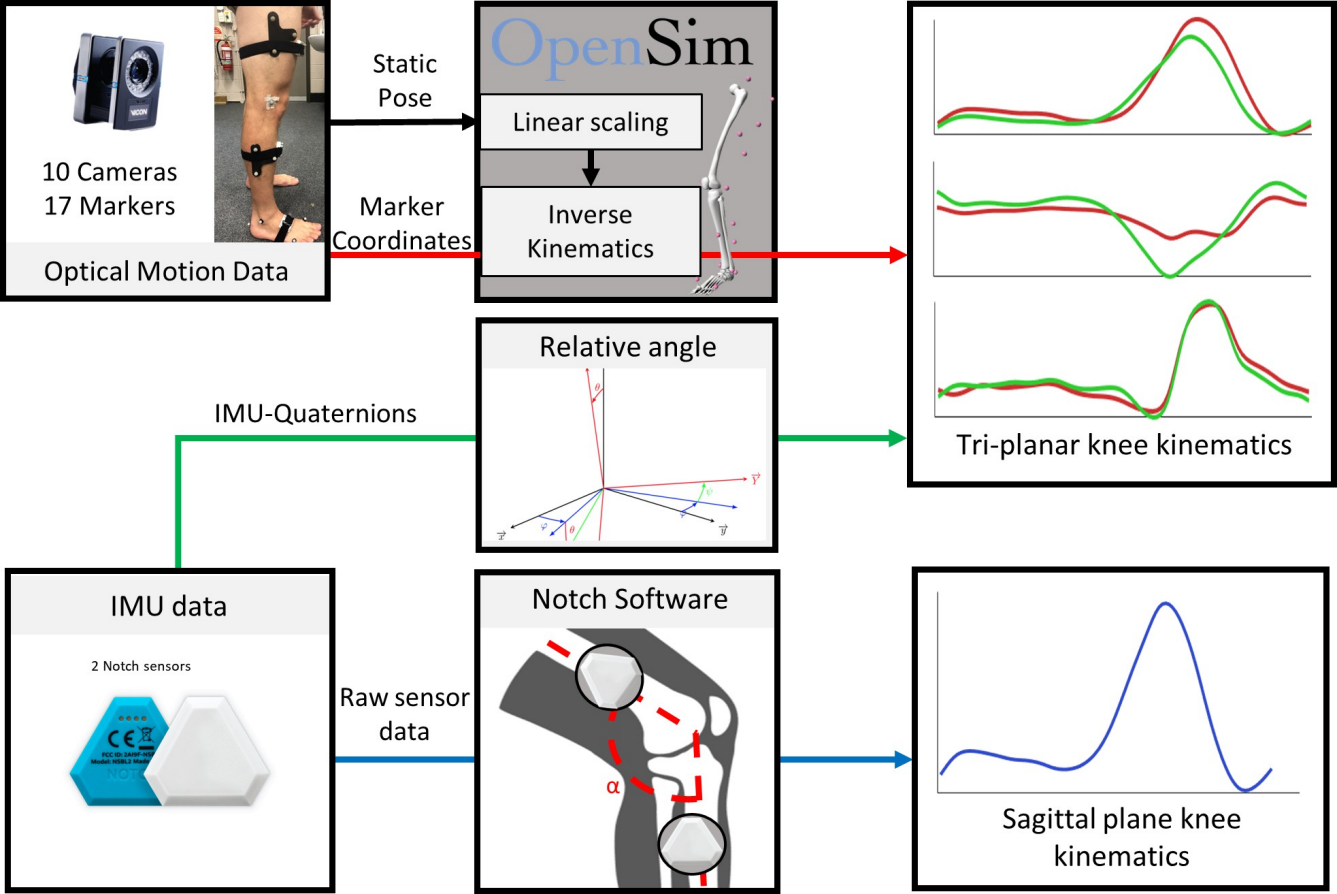
Schematic of the data processing pipeline. Marker trajectories from optical motion capture and accelerations and rotations captured from the Notch IMU system (m_IMU) were recorded simultaneously during 5 activities of daily living. Tri-planar knee angles of were calculated using inverse kinematics analysis in OpenSim using marker trajectories (OMC) and from direct calculation from quaternions (r_IMU). Only sagittal plane knee angles were calculated for IMU system with embedded kinematic model (m_IMU) as depicted in the bottom right-hand plot.

Knee joint angles and ROM were calculated for each task using three methods: (i) OMC driven inverse kinematics in Opensim (OMC); (ii) direct calculation of relative angles from raw quaternion outputs of IMU (r_IMU); and by (iii) a proprietary IMU-driven kinematic model (Wearnotch, Notch Interfaces,Inc., NJ, USA) (m_IMU) (Fig 2). Ground reaction forces were used solely to identify gait events as well as the beginning and end of the squat and sit-to-stand movements. Marker trajectories and ground reaction forces were filtered using a second-order low-pass Butterworth filter with a cut-off frequency of 6 Hz prior to model scaling and inverse kinematics analysis. Marker positions from each participant’s static relaxed standing trials were used to linearly scale a generic 3-segment (thigh, shank, foot) musculoskeletal model with 3 degrees of freedom (sagittal, frontal, and transverse rotations) at both the knee and ankle in OpenSim v4.1 [34, 35]. Scaled models for each participant were subsequently used for all inverse kinematics in the OMC analysis.

For the r_IMU method, the three-dimensional relative motions between the thigh and shank were computed using the raw quaternions exported from the IMUs on-board sensor fusion algorithm. These quaternions were registered to the model during the calibration pose to determine the sensor to segment-to-segment alignment. Quaternions were converted to rotation matrixes, which were used to calculate the change in angle between the shank and thigh segments, prior to conversion to Euler angles using a XYZ sequence. For the OMC, marker trajectories were used in OpenSim inverse kinematics to compute flexion-extension, adduction-abduction, and internal-external rotation of the knee. For r_IMU method, joint angles were calculated as the rotation from thigh orientation to the shank orientation. The Notch IMU system automatically filtered and optimised raw data to calculate segment orientations. The Notch system computed sagittal knee joint angles only, using the measured orientations from the thigh and shank IMUs and Notch proprietary software. Thus, only the r_IMU method was analysed and compared to OMC for frontal and transverse plane motion.

A single representative trial (clear heel-strike/toe-off on force plates, no use of safety rail, clear start and end to squat and sit-to-stand movement, and no marker occlusion) for each participant was analysed for each task. Kinematic data from each method were time aligned using cross-correlation and spline interpolated to 101 time-points. To reduce errors associated with incorrect posture during calibration (i.e., standing with flexed knee), the relative angles were compared by subtracting the mean angle of each trial from all values, for each system. ROM was calculated as the maximum angle minus the minimum angle for each movement cycle.

### 2.5 Statistical analysis

Knee angles across the movement cycle and ROM using r_IMU and m_IMU methods were compared to OMC using Pearson’s correlation coefficient (R) and RMSD. R scores range between 0 and 1 and were stratified as: poor similarity (0 to 0.60); moderate (0.60 to 0.75); good (0.75 to 0.85); very good (0.85 to 0.95); and excellent (0.95 to 1) [36]. Sagittal plane knee ROM was calculated for each system and compared using a repeated measures ANOVA (P < 0.05). For significant differences, post-hoc paired t-tests with Bonferroni correction were performed. Pearson’s correlation coefficients and RMSD were computed in MATLAB v2022a, whilst the repeated measures ANOVA was conducted using SPSS v28.

## Results

### 3.1 Correlation and RMSD–sagittal plane

**Fig 3:**
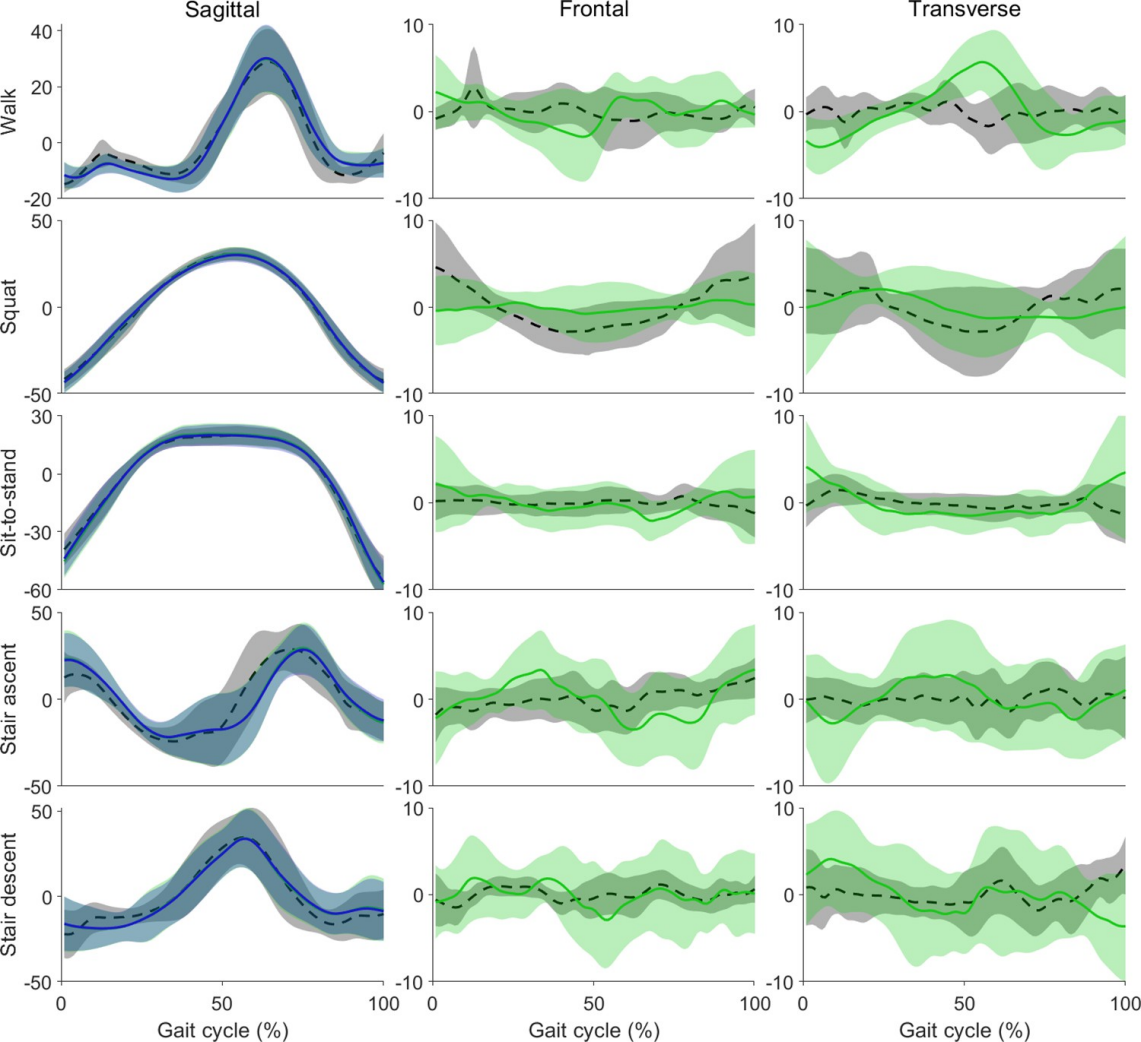
Comparison of IMU measured knee kinematics to optical motion capture. Group mean (± 95% confidence interval) time-series normalised knee kinematics during walking (n = 15), squatting (n = 17), sit-to-stand (n = 17), stair ascent (n = 12), and stair descent (n = 11) from OMC (grey dashed lines), r_IMU (green solid lines), and the m_IMU (blue solid lines) methods. Positive values indicate flexion, adduction, and internal rotation.

In the sagittal plane the m_IMU and r_IMU methods estimated knee angles across the entire movement with excellent correlation (>0.95) compared to OMC method for walking, squatting, sit-to-stand, and stair-ascent, and very good correlation (>0.90) for stair descent (Table 1, Fig 3). The task with the highest correlation and lowest RMSD for the m_IMU was squatting (R = 1.00±0.01, RMSD = 3.42±1.53), followed by sit-to-stand (R = 0.98±0.04, RMSD = 4.19±1.91), walking (R = 0.93±0.09, RMSD = 6.16±2.94), stair ascent (R = 0.88±0.11, RMSD = 11.95±5.30), and stair descent (R = 0.88±0.13, RMSD = 11.81±8.38). Similarly, the r_IMU method demonstrated highest correlation and lowest RMSD for squatting (R = 0.99±0.01, RMSD = 3.94±2.24), followed by sit-to-stand (R = 0.98±0.04, RMSD = 4.57±1.90), walking (R = 0.93±0.08, RMSD = 6.19±2.90), stair ascent (R = 0.88±0.11, RMSD = 12.06±5.21), and stair descent (R = 0.88±0.13, RMSD = 11.81±8.38).

**Table 1:** Group mean (± standard deviation) Pearson’s correlation coefficient (R) and root-mean-square-difference (RMSD, in degrees) of the m_IMU and r_IMU calculated knee kinematics compared to the OMC.

Task	Method	R	RMSD
		mean (SD)	mean (range)
**Walk**	**m_IMU flexion-extension**	0.94 (0.08)	5.77 (3.14–13.28)
**n = 14**	**r_IMU flexion-extension**	0.94 (0.08)	5.76 (3.23–12.68)
	**r_IMU adduction-abduction**	0.44 (0.26)	7.00 (2.69–15.82)
	**r_IMU internal external rotation**	0.44 (0.27)	6.81 (2.96–12.47)
**Squat**	**m_IMU flexion-extension**	1.00 (0.01)	3.42 (1.10–6.45)
**n = 17**	**r_IMU flexion-extension**	0.99 (0.01)	3.94 (1.08–10.12)
	**r_IMU adduction-abduction**	0.69 (0.23)	6.49 (2.19–13.11)
	**r_IMU internal external rotation**	0.78 (0.15)	6.28 (2.19–14.38)
**Sit-to-stand n = 17**	**m_IMU flexion-extension**	0.98 (0.04)	4.19 (1.25–8.71)
	**r_IMU flexion-extension**	0.98 (0.04)	4.57 (1.37–8.77)
	**r_IMU adduction-abduction**	0.51 (0.31)	5.89 (1.58–14.49)
	**r_IMU internal external rotation**	0.60 (0.29)	5.19 (2.06–10.79)
**Stair ascent n = 12**	**m_IMU flexion-extension**	0.88 (0.11)	11.95 (3.51–21.24)
	**r_IMU flexion-extension**	0.88 (0.11)	12.06 (3.95–21.67)
	**r_IMU adduction-abduction**	0.50 (0.26)	6.78 (3.67–13.79)
	**r_IMU internal external rotation**	0.42 (0.28)	7.31 (3.54–13.09)
**Stair Descent n = 11**	**m_IMU flexion-extension**	0.88 (0.13)	11.81 (2.47–26.15)
	**r_IMU flexion-extension**	0.88 (0.13)	11.79 (2.48–24.99)
	**r_IMU adduction-abduction**	0.40 (0.18)	6.69 (3.85–13.10)
	**r_IMU internal external rotation**	0.45 (0.31)	7.73 (3.85–15.72)

The two IMU methods were compared to optical motion capture for each task. In the sagittal plane, both IMU methods had excellent correlation to OMC for squatting and sit-to-stand, and very good correlation for walking stair ascent and stair descent. The lowest RMSD was for squatting, followed by sit-to-stand, walking, stair descent, and stair ascent. For transverse and frontal planes, the correlation to OMC for both IMU methods was poor to moderate and had RMSD larger than the range-of-motion. R = Pearson’s correlation coefficent, RMSD = root-mean-square-error, SD = standard deviation.

### 3.2 Correlation and RMSD–frontal and transverse planes

In the frontal plane, the r_IMU estimated knee kinematics with poor (R < 0.60 for walking, sit-to-stand, stair ascent, and stair descent) to moderate (R = 0.6 to 0.75 for squatting) correlation with OMC values (Table 1, Fig 3). The highest R (0.69±0.23) and lowest RMSD (5.89±63.80) for the frontal plane were found in the squatting and sit-to-stand tasks, respectively. In the transverse plane, the r_IMU estimated knee kinematics with poor (R < 0.60 for walking, stair ascent, and stair descent), moderate (R = 0.6 to 0.75 for sit-to-stand) and good (R = 0.75 to 0.85 for squatting) correlation with OMC values (Table 1, Fig 3). For the transverse plane, the highest R (0.78±0.15) was found in the squatting task whilst the lowest RMSD (5.19±2.94) was found in walking. The errors observed in the frontal and transverse planes are high considering the low ROM observed in these planes (Fig 3).

### 3.3 Range of motion–sagittal plane

Between the three methods, there was no significant difference in sagittal plane ROM for walking, squatting, and sit-to-stand tasks (Table 2). For stair ascent, a repeated measures ANOVA found a significant difference between the OMC (88.56±9.72), m_IMU (84.93±12.19), and r_IMU (82.42±11.70) (F = 5.37, p = 0.03). For stair descent, there was a significant difference between OMC (88.90±9.63), m_IMU (83.46±9.76), and r_IMU (81.72±9.35) (F = 4.95, p = 0.04). However, post-hoc comparison with Bonferroni correction (*p<0.017) showed no significant difference between methods for both stair ascent and stair descent.

**Table 2:** Group mean (± standard deviation) knee sagittal plane range of motion (ROM) and mean difference between methods (95% confidence interval) calculated using optical motion capture (OMC), model IMU (m_IMU) and raw IMU (r_IMU) data. r_IMU.

Task	Method	Range of motion	Mean difference
		mean (SD)	(95% CI)
**Walk**	**OMC**	60.40 (8.77)	2.14 (-3.01,7.28)^a^
**n = 14**	**m_IMU**	58.26 (6.61)	1.92 (-3.57,7.40)^b^
** **	**r_IMU**	58.48 (6.70)	-0.22 (-1.28,0.84)^c^
**Squat**	**OMC**	78.85 (11.73)	-0.97 (-6.57,4.63)^a^
**n = 17**	**m_IMU**	79.82 (13.13)	0.03 (-6.64,6.71)^b^
** **	**r_IMU**	78.81 (13.20)	1.00 (-1.42,3.42)^c^
**Sit-to-stand**	**OMC**	77.42 (23.51)	-4.78 (-10.44,0.88)^a^
**n = 17**	**m_IMU**	81.24 (27.58)	-2.71 (-9.71,4.29)^b^
** **	**r_IMU**	79.27 (28.85)	2.07 (-1.61,5.74)^c^
**Stair ascent** *	**OMC**	88.56 (9.72)	3.64 (-2.29,9.56)^a^
**n = 12**	**m_IMU**	84.93 (12.19)	6.14 (-0.30,12.58)^b^
** **	**r_IMU**	82.42 (11.70)	2.51 (-0.34,5.35)^c^
**Stair Descent** *	**OMC**	88.90 (9.63)	5.44 (-2.95,13.83)^a^
**n = 11**	**m_IMU**	83.46 (9.76)	7.18 (-0.58,14.94)^b^
	**r_IMU**	81.72 (9.35)	1.74 (-1.36,4.84)^c^

A repeated measures ANOVA was performed to compare mean sagittal plane range of motion (ROM) between the three methods, finding a significant difference for stair ascent and stair descent. Post-hoc pair-wise comparison with Bonferroni correction showed no significant difference between methods, with 95% confidence interval for mean difference for all comparisons crossing zero.

*p<0.05, CI = confidence interval, SD = standard deviation

^a^ = OMC—m_IMU

b = OMC—r_IMU

c = m_IMU—r_IMU.

## Discussion

Inertial measurement units provide an easy-to-use and objective tool to assess knee function and recovery following TKA. In participants 1-year post robotically assisted TKA, we found IMU methods (m_IMU and r_IMU) could measure sagittal plane knee angles with very good to excellent correlation across five activities of daily living (walking, squatting, sit-to-stand, stair ascent, and stair descent) compared to OMC, in agreement with our first hypothesis. For example, walking showed excellent correlation and mean RMSD of less than 6.5 degrees compared to OMC. The highest correlation and lowest RMSD were observed during squatting and sit-to-stand, suggesting that IMU-based estimations better match OMC-based estimations during closed-chain, low velocity movements. Frontal and transverse plane knee kinematics calculated using IMU methods, should however be interpreted with caution, as they typically demonstrated lower correlations and higher RMSD relative to their total ROM compared to OMC across all tasks. Differences between the three methods were identified in ROM for stair ascent and stair descent tasks only, although pairwise comparison failed to identify significant difference between comparisons, suggesting differences are not meaningful. Therefore, our findings support the use of IMU as a clinical tool for measurement of sagittal plane knee kinematics and ROM during activities of daily living in individuals following TKA. The implementation of functional assessments should, however, consider tasks with excellent correlation and low error compared to gold standard measurement such as squatting, sit-to-stand, and walking. IMU offer an easy-to-use, rapid, and cost-effective method for sagittal plane knee kinematics analysis in the clinic, outdoors, or in the home where OMC is impractical.

While minimum differences were found between the r_IMU and m_IMU methods, an IMU system with an underlying kinematic model (m_IMU) may be better suited to clinical applications. IMU systems with an underlying kinematic model and user-friendly software simplify data acquisition (~ 5 mins) and overcome many of the challenges of recording biomechanical data outside a research laboratory [37]. In addition, the real-time kinematic model provides instant feedback on knee function that can be used to assess improvements, identify tasks with suboptimal biomechanics, and guide rehabilitation exercises. Knee flexion ROM, an important clinical measure indicative of recovery following TKA [38], is typically measured passively or actively using goniometers [39]. Assessment during functional tasks, like walking, offers clinically relevant insight of knee function following TKA [40, 41]. A potential application is the use of IMU at scale to elucidate differences in functional outcomes due to surgical techniques, surgery duration, implant positioning, and type of implant, and may be enhanced after robotically assisted TKA during which surgical data may be automatically logged.

These results are consistent with previous studies that estimated sagittal plane knee angles during walking with RMSD between 4–7 degrees [30, 42, 43]. The differences observed may be attributed to several factors including gyroscopic drift for IMUs [43], a non-zero knee angle during the calibration pose, and movement of the soft tissue to which the sensors and markers are fixed [44]. The magnitude of soft-tissue artefact is affected by the location of markers/sensor placement, with studies suggesting the thigh is particularly susceptible to translational and rotational errors [45, 46]. Soft tissue artefact is also dependent on individual characteristics [46] and may be greater in participants with high levels of adipose tissue. Additionally, soft-tissue artefact may affect IMU and OMC methods differently, with a recent study showing a IMU method over-estimated sagittal plane knee angles during stance, and underestimated them during swing [47]. Characterising the differing effects of soft-tissue artefact on data collection systems will allow for the development of correction methods that will improve IMU-based predictions. Finally, although OMC is considered the gold-standard for non-invasive human motion capture, error in the OMC method should also be considered as a source of difference, with a comparison of five marker-based OMC protocols showing a mean absolute variance of greater than 3 degrees between protocols [47]. Additionally, the mean RMSD in the flexion-extension axis for both the m_IMU and r_IMU methods when compared to OMC were within the reported uncertainty for OMC-based kinematic measurements for all tasks except stair ascent and stair descent [48].

There are limitations in our study that should be considered. The r_IMU method measured frontal and transverse plane kinematics with poor to moderate correlation to the OMC method. The disagreement in the frontal and transverse planes may be attributed to sensitivity to marker placement protocols [47], the high ratio of soft-tissue movement to joint motion in the frontal and transverse planes, and the definition of the flexion-extension axis [49]. Errors in the flexion-extension axis alignment during calibration can lead to angular crosstalk, where motion is incorrectly represented in the transverse and frontal planes. While our results support the use of IMU for the measurement of sagittal plane knee kinematics across a range of ADL, we did not assess reliability during long recordings, for which orientation estimates may drift, and error can increase [42]. Additionally, we used embedded force plates (ground and instrumented staircase) to identify gait events which may affect IMU orientations due to electromagnetic interference [50]. Greater angular crosstalk due to more time spent in knee flexion compared to walking, and greater magnetic electromagnetic disturbance of the largely exposed instrumented staircase likely contributed to poorer results in the stair ascent and descent tasks. Further, several participants were excluded from analysis due to marker occlusion, particularly in stair-related tasks caused by the required safety railings. There may be differences in r_IMU and m_IMU range of motion compared to OMC that were not detected due to our small sample size. Nevertheless, we believe the high correlation and low RMSD in the flexion-extension axis supports the use of IMUs for measurement of sagittal plane knee kinematics during ADL. To ensure participants could complete multiple tasks in a single session, participant inclusion criteria included the ability to walk for 30 minutes continuously and unaided. Excluding participants who could not walk for 30 minutes continuously likely biased our study to individuals with good TKA outcomes who may have different knee kinematics to those with poor TKA outcomes. Future studies may build on our findings by assessing the reliability of IMU-based measurements of kinematics and ROM during ADL in the real-world (e.g., walking outside, functional tasks in the home), assess the ability for IMU to detect clinically meaningful changes in knee kinematics, and prospectively evaluate changes in knee kinematics post TKA over the long term. Additionally, future studies may improve results in the frontal and transverse planes by utilising functional calibration tasks and using biplanar radiography to assess the frontal and transverse accuracy of IMU measured knee kinematics [51]. Estimation of knee kinematics may also be improved by combining IMUs with marker-less motion capture [52], body-worn cameras [50] or machine learning techniques [53, 54], which have shown promising results in predicting human movement kinematics.

## Conclusions

Our results demonstrate that IMU may accurately quantify sagittal plane knee motion across various ADL that may complement subjective patient reported outcome measures throughout the episode of care for TKA patients. Both IMU methods have excellent agreement with gold-standard OMC methods for sagittal plane knee kinematics during squatting, sit-to-stand, and walking. Frontal and transverse plane kinematics measured with IMU showed poor-to-moderate correlation with OMC and should be interpreted with caution. Objective measures of knee kinematics may provide insight into the effectiveness of surgical techniques and can be measured easily and cost-effectively using IMU. Future studies should determine if knee kinematics from IMU measurements can discriminate between excellent, moderate, and poor outcomes, and to what extent these differences are associated with surgical techniques.

## Supporting information

S1 Checklist STROBE statement—checklist of items that should be included in reports of observational studies. (DOCX)

S1 Dataset Time-series knee angles measured by OMC and IMU. (CSV)
